# Quantitative proteomics identification of phosphoglycerate mutase 1 as a novel therapeutic target in hepatocellular carcinoma

**DOI:** 10.1186/1476-4598-9-81

**Published:** 2010-04-19

**Authors:** Fenglian Ren, Hong Wu, Yunlong Lei, Haiyuan Zhang, Rui Liu, Yong Zhao, Xiancheng Chen, Dequan Zeng, Aiping Tong, Lijuan Chen, Yuquan Wei, Canhua Huang

**Affiliations:** 1 The State Key Laboratory of Biotherapy, West China Hospital, Sichuan University, Chengdu, 610041, China; 2 Department of Hepatobiliary Pancreatic Surgery, West China Hospital, Sichuan University, Chengdu, 610041, China; 3 The School of Medicine, Yangtze University, Shashi, Hubei, 434000, China

## Abstract

**Background:**

Hepatocellular carcinoma (HCC) is one of the most common malignancies worldwide with poor prognosis due to resistance to conventional chemotherapy and limited efficacy of radiotherapy. There is an urgent need to develop novel biomarkers for early diagnosis, as well as to identify new drug targets for therapeutic interventions.

**Patients and methods:**

54 paired HCC samples and 21 normal liver tissues were obtained from West China Hospital of Sichuan University. Informed consent was obtained from all the patients or their relatives prior to analysis, and the project was approved by the Institutional Ethics Committee of Sichuan University. Stable Isotope Labeling with Amino Acids in Cell Culture (SILAC)-based proteomics was employed to profile the differentially expressed proteins between a HepG2 human hepatoma cell line and an immortal hepatic cell line L02. Validation of PGAM1 expression was performed by semi-quantitative RT-PCR, immunoblot and immunohistochemistry using clinical samples. shRNA expressing plasmids specifically targeting PGAM1 were designed and constructed by GenePharma Corporation (Shanghai, China), and were utilized to silence expression of PGAM1 *in vitro *and *in vivo*. Cell proliferation was measured by a combination of colony formation assay and Ki67 staining. Apoptosis was examined by flow cytometry and TUNEL assay.

**Results:**

A total of 63 dysregulated proteins were identified, including 51 up-regulated proteins, and 12 down-regulated proteins (over 2-fold, *p *< 0.01). Phosphoglycerate mutase 1 (PGAM1) was found markedly upregulated. Clinico-pathological analysis indicated that overexpression of PGAM1 was associated with 66.7% HCC, and strongly correlated with poor differentiation and decreased survival rates (*p *< 0.01). shRNAs-mediated repression of PGAM1 expression resulted in significant inhibition in liver cancer cell growth both *in vitro *and *in vivo*.

**Conclusion:**

Our studies suggested that PGAM1 plays an important role in hepatocarcinogenesis, and should be a potential diagnostic biomarker, as well as an attractive therapeutic target for hepatocellular carcinoma.

## Background

Hepatocellular carcinoma (HCC) is the fifth most common malignancy worldwide with poor prognosis, and is responsible for 600 000 deaths annually worldwide [1-5]. Many patients are diagnosed at the advanced stage and missed the best opportunity for effective therapy, such as liver resection, or transplantation. On the other hand, patients who were resected often have a high frequency of metastasis/recurrence, and postoperative 5-year survival is only 30%-40% [6]. Moreover, liver transplantation is not applicable universally because of the shortage of organ donations and occurrence of relapse [7]. Consequently, there is an urgent need to screen for novel therapeutic targets.

The metabolism of cancer cells differs significantly from that of normal cells [8]. Cancer cells are able to maintain high rates of aerobic glycolysis even under the high-oxygen (20%) conditions of normal tissue culture. This property, known as the "Warburg effect", has been recognized for over 70 years [9,10]. In this context, maintaining a high level of glycolysis is indispensable for survival and growth of cancer cells [11]. Guided by this principle, intervention with cellular glucose utilization could lead to a significant inhibition of cell growth, induction of cell death, stimulating migration of key enzymes out of the glycolytic enzyme complexes as well [12,13]. Recently, chemistry-based functional proteomics was applied to screen for drug target against breast cancer, and phosphoglycerate mutase 1 (PGAM1) was identified as a novel metabolic enzyme involved in breast carcinogenesis [14].

In adult mammals, three isozymes of PGAM are present which result from the homo- and heterodimeric combinations of two different 30-kD subunits, M and B, encoded by two different genes [15,16]. The homodimer BB-PGAM (a brain form; PGAM1 in human), is expressed mainly in liver, kidney, and brain; the homodimer MM-PGAM (muscle-specific form; PGAM2 in human), is mainly found in the mature muscle cells; and the heterodimer MB-PGAM, mainly exists in heart [16-18]. Particularly, PGAM1, a key enzyme of the glycolytic pathway, converts 3-phosphoglycerate to 2-phosphoglycerate with 2, 3-bisphosphoglycerate (2, 3-BPG) as a cofactor of the reaction to release energy which is essential for cell growth [19]. Several investigations demonstrated that PGAM1 was overexpressed in a variety of human cancers, including breast carcinoma [14,20]; colorectal cancer [21,22]; lung cancer [23,24]; prostate cancer [19]; oral squamous cell carcinoma [25]; esophageal squamous cell carcinomas [26]; and also associated with certain virus infection [27,28]. Overexpression of PGAM1 can immortalize mouse embryonic fibroblasts and promote cell proliferation, suggesting its potential oncogenic property [10]. Furthermore, a recent study showed that a PGAM1 peptide inhibitor induced cancer cell growth arrest in breast carcinoma [14]. Taken together, targeting the PGAM1 may be preferentially lethal to the malignant cells and have potentially broad clinical and therapeutic implications.

In the present study, we utilized a quantitative proteomic approach to profile the altered expressed proteins between a liver cancer cell line HepG2, and an immortalized human normal hepatocyte cell line L02. Of the 63 dysregulated proteins, we found that PGAM1 was significantly upregulated. Clinicopathological analyses revealed that overexpression of PGAM1 was closely associated with hepatocarcinogenesis. The data presented in this study suggested that PGAM1 could be developed as a useful diagnostic biomarker, as well as a potential therapeutic target for hepatocellular carcinoma.

## Experimental Procedures

### Clinical specimens

Paraffin-embedded HCC and the adjacent normal tissues were obtained from 54 patients who underwent surgical resections at the West China Hospital of Sichuan University (Chengdu, China). Histodifferentiation grading of specimens was assigned according to Edmondson Steiner grading by experienced pathologists. 21 normal liver specimens were collected from patients undergoing surgical resections for hepatic cyst or calculus of intrahepatic duct. The surgical pathologic staging was assigned according to the modified UICC classification [29]. A summary of detailed clinicopathologic information for these patients was shown in Table 1. Each tissue was cut into two parts: one part was snap frozen for immunoblot and RT-PCR validation, and the other part was fixed in formalin for immunohistochemistry analysis. Informed consent was obtained from all the patients or their relatives prior to analysis, and the project was approved by the Institutional Ethics Committee of Sichuan University.

**Table 1:** Clinicopathologic features of all patients.

Clinicopathologic Features	Number	**Mean Age**^**a**^**(%)**
Normal liver tissues	21	45.4 ± 5.7
Hepatocellular carcinoma	54	44.8 ± 8.5 (100.0)
Histodifferentiation grading		
Well differentiated	11	45.2 (20.4)
Moderately differentiated	28	40.6 (51.9)
Poorly differentiated	15	46.4 (27.8)
Surgical pathologic staging		
I	14	48.1 (25.9)
II	21	43.9 (38.9)
III	9	46.1 (16.7)
IV	10	45.3 (18.5)

^a ^Mean age in years.

### Cell culture, SILAC labeling and transfection

Human hepatoma cell line HepG2 was purchased from ATCC (Rockville, MD, USA) and maintained in Dulbecco's modified Eagle's medium (DMEM, Gibco, USA) supplemented with 10% fetal calf serum (Hyclone, USA), penicillin (10^7^U/L) and streptomycin (10 mg/L) at 37°C in a 5% CO_2 _atmosphere. Human immortalized normal liver cell line L02 was obtained from China Cell Culture Center (Shanghai, China) and cultured in Dulbecco's modified Eagle's medium (DMEM, Gibco, USA) [30].

For SILAC-labelling, cells were grown in SILAC™ D-MEM (Invitrogen, USA) containing 10% v/v dialysed FBS, 2 mM L-glutamine, and either 0.1 mg/mL SILAC™ light [^12^C_6_] or SILAC™ heavy [^13^C_6_] L-lysine (Invitrogen). To ensure full incorporation of the heavy and light labeled amino acids, cells were grown for at least six cell doublings prior to analysis.

For transient transfection, HepG2 cells were seeded in 6- or 96-well culture plates at a density of 10^5 ^cells or 5000 cells/well, respectively. After incubated overnight, cells were transfected with PGAM1-shRNA (2 μg/well) or the scramble shRNA using Lipofectamine 2000 (Invitrogen, Carlsbad, CA) according to the manufacturer's instructions.

### Protein preparation and SDS-PAGE separation

Equal amounts of protein from HepG2 (^13^C_6_-lysine) and L02 (^12^C_6_-lysine) cell lines were mixed (60 μg in total), boiled in SDS-PAGE sample buffer, resolved by SDS-PAGE and stained with Coomassie Brilliant Blue R-250 (Merck, Germany).

### In-gel trypsin digestion

In-gel digestion was performed using mass spectrometry grade Trypsin Gold (Promega, Madison, WI, USA) according to the manufacturer's instructions. Briefly, the excised gel slices were destained twice with 100 mM NH_4_HCO_3_/50% acetonitrile at 37°C for 45 min, and dried in a centrifugal vacuum concentrator, followed by a preincubation in 10-20 μl trypsin solution for 1 h. Subsequently the digestion buffer were added (40 mM NH_4_HCO_3_/10% ACN) to cover the gel pieces and incubated overnight at 37°C. Tryptic digests were extracted twice with 50% ACN/5% trifluoroacetic acid (TFA) for 1 h each time. The combined extracts were dried in a vacuum concentrator to a final volume of 5 μl at room temperature. The samples were then subjected to mass spectrometric analysis.

### Protein identification and quantitation by LC-MS/MS

Mass spectra were acquired using a LC-MS mass spectrometer (Micromass, Manchester, UK). Tryptic digests were dissolved in 20 μl of 50% ACN. The automatic scan rate was 1.0 s with an interscan delay of 0.02 s, and the voltage was operated at 3.0 KV. Spectra were accumulated until a satisfactory signal/noise ratio had been obtained with the range 400 - 1600 m/z picked out for LC-MS/MS analysis. The collision energy was varied between 18 - 57 eV depending on the mass of the precursor. Quantitation was carried out by SILCA K+ 6 R+ 10 [MD]. The MS/MS data, "pkl list" files were acquired by the software ProteinLynx 2.2.5 software (Waters), which include the mass values, the intensity and the charge of the precursor ions (parent ions with +1, +2 or +3 charges in this study). The pkl files were analyzed using the MASCOT search engine against the Swiss-Prot protein database. The search parameters were carried out as follows: Database, Swiss-Prot; taxonomy, *homo sapien*; enzyme, trypsin; and an allowance of one missed cleavage. Carbamidomethylation was set as a fixed modification and oxidation of methionine was variable. The peptide and fragment mass tolerance were both set at 0.2 Da. Proteins were identified at least one peptide exceeding their score threshold (*p *< 0.01) and with their MW and pI consistent with the gel regions from which the bands were excised indicated the 95% confidence level for the matched peptides. Protein intensity alteration (>2 fold) was defined as dysregulation.

### Semi-quantitative RT-PCR

The following primer sequences were used to detect PGAM1 transcripts: sense (5'-GCACCCACTCCCTTCATACAAT-3') and antisense (5'-ACGCAGGTTACATTCGTCTTCC-3'). Total RNA was extracted using Trizol Reagent (Invitrogen, USA). RT-PCR reaction was performed as follows: reverse transcription at 45°C for 30 min and denaturation at 94°C for 2 min; then amplification for 30 cycles at 94°C for 30 s, annealing at 54°C for 1 min, and extension at 72°C for 1.5 min, followed by a terminal elongation step at 72°C for 10 min and a final holding stage at 4°C. PCR products were resolved by 1% agarose gel electrophoresis.

### Western blotting analysis

Western blotting analysis was performed as described elsewhere [31]. Briefly, 30 μg of proteins were separated by 12% SDS-PAGE and transferred to PVDF membranes (Amersham Biosciences). After blocking overnight with TBS/T containing 0.1% Tween 20 in 5% skimmed milk at 4°C, the membranes was subsequently probed with primary antibody PGAM1 (diluted 1:1000, Abcam) for 2 h at RT and washed 3 times in TBS/T. Subsequently the membranes were incubated with secondary antibody conjugated to Horseradish Peroxidase for 2 h at RT. Immunoblot was detected by the enhanced chemiluminescence (ECL) detection system (Pierce Biotech Inc., Rockford, IL, USA).

### Immunohistochemistry analysis

Tissues were formalin-fixed and Paraffin-embedded, and sections were consecutively cut into 3-4 μm thickness for immunohistochemistry (IHC) analysis using a Dako EnVision System (Dako Cytomation GmbH, Hamburg, Germany Denmark) according to the manufacturer's instructions. Briefly, the paraffin-sections were dewaxed, rehydrated, and incubated in 3% H_2_O_2 _for 10 min in dark at room temperature to quench the endogenous peroxidase activity. Antigen retrieval was performed in citrate buffer (pH 6.0) using autoclave sterilizer method. Subsequently, the sections were blocked by normal rabbit or goat serum diluted in PBS (pH 7.4) for 20 min at 37°C, followed by an incubation at 4°C overnight with the primary antibodies, either goat anti-PGAM1 (diluted 1:150, Abcam, Cambridge, UK) or mouse anti-Ki67 (diluted 1:200, Santa Cruz Biotechnology, Santa Cruz, CA). After rinse in fresh PBS for 15 min, slides were incubated with horseradish peroxidase-linked rabbit anti-goat and anti-mouse antibodies at 37°C for 40 min, followed by reaction with 3,3'-diaminobenzidine substrate solution (Dako Cytomation GmbH) and counterstaining with Mayer's hematoxylin. The immunohistochemical staining was assessed by calculating the percentage of positive hepatocytes and the immunostaining intensity [32]. Slides were examined separately by two independent pathologists with no prior knowledge of each patient's clinical and pathological parameters. Any discrepancy between the two evaluators was resolved by reevaluation and careful discussion until agreement was reached.

### Cell proliferation and apoptosis assay

Upon treatment, cells were incubated at 37°C for indicated durations. Cell proliferation was measured by means of MTT assay according to the manufacturer's instructions. 20 μl MTT (2 mg/ml, Sigma, St. Louis, MO) was added in the media and incubated for another 2 h. The media was removed and formazan precipitate was dissolved in 150 μl Dimethyl Sulfoxide (DMSO, Amresco, Solon, Ohio, USA). Ten minutes later, absorbance values were measured at 595 nm wavelength on a Spectra Max M5 (MDC, Sunnyrale, VA, USA).

For colony formation assay, cells were seeded in 6-well plates at a density of 300 cells per well. Assay was performed at 24 h posttransfection, cells were cultured for another two weeks. Colonies were washed with PBS, fixed with methanol and stained with Crystal Violet (Sigma, St. Louis, MO, USA). Cells were counted under a microscope and a cluster with more than 50 cells was considered as a clone.

For flow cytometric analysis, cells were trypsinized and washed with 0.9% NaCl at 72 h post transfection, and then fixed with 70% ethanol at 4°C overnight. Cells were incubated with staining solution (5 μg/ml propidium iodide, 20 μg/ml Rnase A) in dark at room temprature for 1 h. The stained cells were analyzed on an EPICS ELITE ESP flow cytometer (Beckman Coulter, USA).

TUNEL staining was performed using terminal deoxynucleotidyl transferase (Promega Inc., Madison, WI, USA). Cells were fixed in freshly prepared 4% methanol-free formaldehyde solution in PBS (pH 7.4) for 25 minutes at 4°C, washed with fresh PBS for 10 minutes at room temperature and permeabilized in 0.2% Triton-100 solution in PBS for another 5 min. After equilibration for 10 min, the cells were incubated with rTdT buffer and observed under a fluorescence microscope, and a nucleus with bright green fluorescence staining was recorded as a TUNEL-positive event (Olympus Optical Co, Hamburg, Germany).

### Tumor xenograft model and shRNA treatment

The cDNA sequence of PGAM1 was obtained from Genbank (NM_002629). Three PGAM1-specific short hairpin RNAs (shRNAs) were designed based on the rules as described elsewhere [33]. As shown in Table S1, additional file 1, shRNA expressing plasmids specifically targeting PGAM1 (termed as PGAM1-shRNA-a, -b and -c) were constructed by GenePharma Corporation (Shanghai, China) using pGPU6/GFP/Neo vector. An unrelated shRNA sequence (HK), with no homology to any human gene, was used as a negative control (shNC).

For animal study, 6-8 weeks old female nude mice (BALB/c, 18-20 g body weight) were injected subcutaneously with HepG2 cell suspensions about 2 × 10^6 ^cells/100 μl/mouse in PBS via the right flank. When the tumor diameter reached about 6 mm, the tumor-bearing mice were randomly divided into four experimental groups (n = 5 mice/group): 1) PBS: 100 μl/mouse; 2) LIPO: Lipofectamine 2000 at 12.5 μl/100 μl of PBS; 3) shNC (negative control): 5 μg/100 μl of PBS; 4) PGAM1-shRNA-a: 5 μg/100 μl of PBS. Tail intravenous injections were performed every two days, and the tumor volumes were evaluated by the following formula: tumor volumes (mm^3^) = π/6 × length × width^2^. The tumor growth inhibition in the presence or absence of PGAM1 shRNA has been monitored for 20 days until the mice were sacrificed. The tumor tissues were formalin-fixed and paraffin-embedded for immunohistochemistry. All animals received humane care according to the Institutional Animal Care and Treatment Committee of Sichuan University.

### Statistics

All quantitative data were recorded as mean ± S.D. Comparisons between two groups were performed by Student's *t *test. Differences among multiple groups were assessed by one-way ANOVA analysis, LSD-*t *test. Relevance analysis of ordinal data was performed by cross χ^2 ^test. Statistical significance was defined as *p *< 0.05.

## Results

### Proteomic profiling of the differentially expressed proteins between HepG2 and LO2 by SILAC

**Figure 1:**
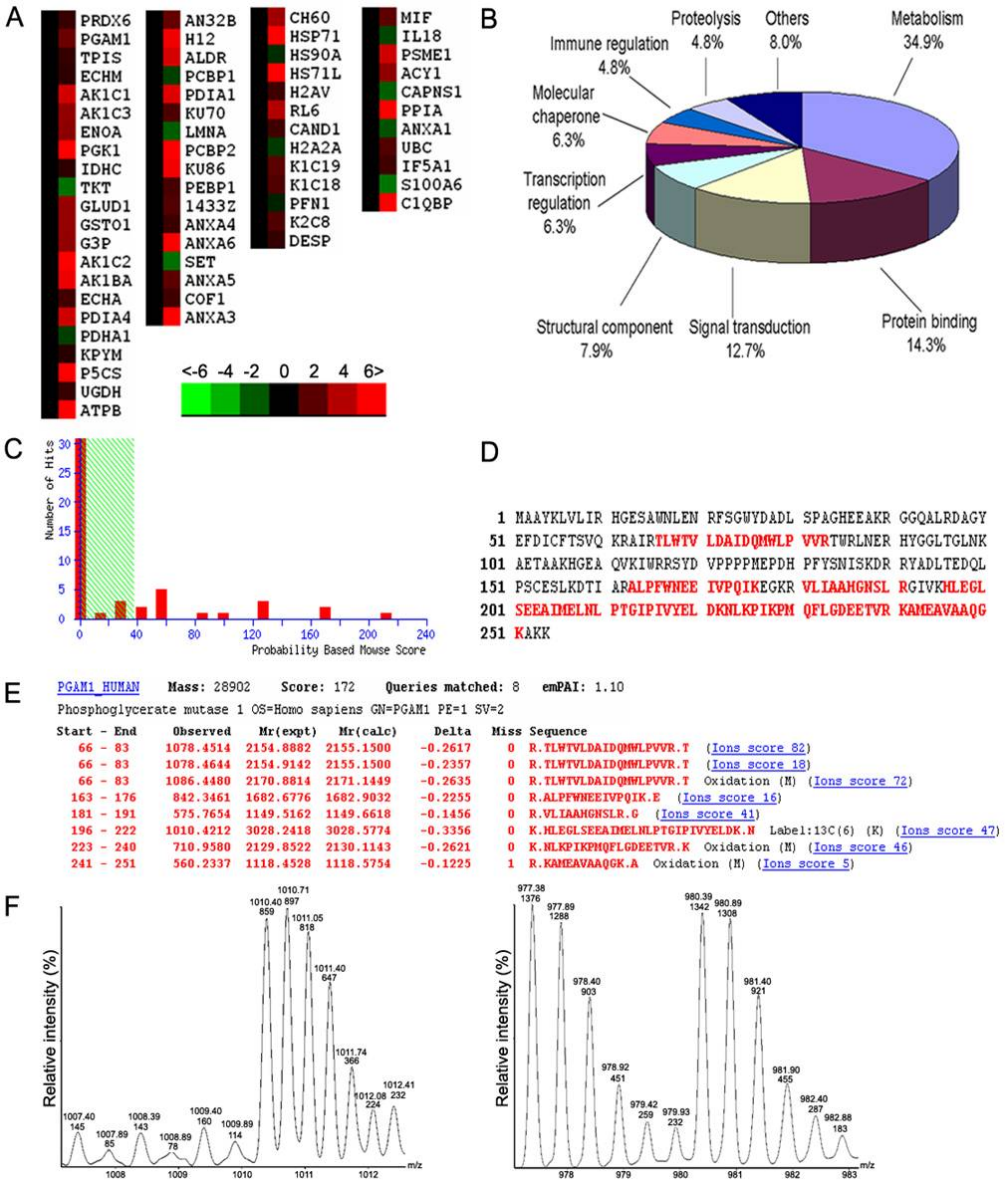
**Identification and quantification of proteins by LC-MS/MS Based on SILAC method**. (A) Clustering analysis of the identified proteins from L02 cells and HepG2 cells. Protein cluster map generated by Cluster software. Expression of proteins in the normal group was constant at 0, proteins up-regulated in cancer tissue were in red, and the down-regulated proteins were in green. The intensity of the color green or red corresponds to the degree of alteration, respectively, according to the color strip at the bottom of the figure. These data were derived from three independent analyses. (B) A total of 63 dysregulated proteins were classified into 11 groups with diverse functions including metabolism (34.9%), signal transduction (12.7%), structural component (7.9%), and other functions (44.5%). (C), (D), and (E) showed output of the LC MS/MS database using the MASCOT program. LC MS/MS analysis revealed 8 matched peptides with 38% sequence coverage and a MOWSE score of 172. The matched peptides were shown in bold red. (F), *left*, MS spectrum showed a SILAC peptide matching PGAM1, with an up-regulation up to 6-fold. The m/z presents a difference of 3 mass units between labeled and unlabeled peptide pair, resulting in a 2^+ ^change state; *right*, control SILAC peptide from β-actin.

Differentially expressed proteins were defined as statistically significant based on two criteria: 1) intensity alterations >2.0-fold (Student's *t *test, *p *< 0.05) and 2) recurrence more than two times in the 3 repeated experiments. According to these criteria, a total of 63 distinct proteins were identified by LC-MS/MS, as listed in Table 2. Cluster analysis revealed that the altered proteins were involved in diverse biological processes, including metabolism (34.9%), signal transduction (12.7%), structural component (7.9%) and others (44.5%) (Fig. 1A and 1B). Among them, PGAM1 was identified with most significant alteration that PGAM1 was up-regulated over 6-fold in HepG2 cells compared to L02 cells (*p *< 0.01). Further, LC MS/MS analysis revealed eight matched peptides, with 38% sequence coverage and a MOWSE score of 172 (Fig. 1, C-F). The housekeeping gene β-actin was always selected to monitor the labeling status.

**Table 2:** Proteins identified by LC MS/MS.

**Protein name**^**a**^	Gene name	**Accession no.**^**b**^	**Theotetical molecular mass/PI**^**c**^	Queries matched	Sequence coverage (%)	**MOWSE score**^**d**^	**Fold change**^**e**^	Function
**Up-regulation**								
Macrophage migration inhibitory factor	MIF	P14174	12642/7.74	1	9	57	3.1 ± 0.9	Immune regulation
Peptidyl-prolyl cis-trans isomerase A	PPIA	P62937	18233/7.68	12	35	238	8.7 ± 2.2	Protein folding
Eukaryotic translation initiation factor 5A-1	IF5A1	P63241	17053/5.08	3	22	214	2.1 ± 0.9	Translation regulation
Histone H2A.V	H2AV	Q71UI9	13501/10.58	2	28	138	3.9 ± 1.1	Transcription regulation
Phosphatidylethanolamine-binding protein 1	PEBP1	P30086	21160/7.01	2	18	92	2.9 ± 0.9	Signal transduction
Peroxiredoxin-6	PRDX6	P30041	25135/6.00	8	40	175	3.1 ± 1.2	Metabolism
Phosphoglycerate mutase 1	PGAM1	P18669	28902/6.67	8	38	172	6.0 ± 1.4	Metabolism
14-3-3 protein zeta/delta	1433Z	Q6P3U9	27902/4.73	9	33	126	3.3 ± 1.2	Signal transduction
Triosephosphate isomerase	TPIS	P60174	26943/6.45	21	45	406	2.9 ± 0.8	Metabolism
Proteasome activator complex subunit 1	PSME1	Q06323	28879/5.78	1	7	59	6.7 ± 2.1	Proteolysis
Enoyl-CoA hydratase, mitochondrial	ECHM	P30084	31831/8.34	1	7	51	2.6 ± 0.8	Metabolism
Annexin A4	ANXA4	P09525	36092/5.84	1	5	98	2.4 ± 0.5	Signal transduction
Annexin A5	ANXA5	P08758	35972/4.94	2	8	52	3.7 ± 1.5	Signal transduction
Aldo-keto reductase family 1 member C1	AK1C1	Q04828	37229/8.02	4	24	109	11.7 ± 2.7	Metabolism
Aldo-keto reductase family 1 member C3	AK1C3	Q9UKL9	37227/8.05	2	10	58	8.1 ± 2.4	Metabolism
Alpha-enolase	ENOA	Q6GMP2	47487/7.01	20	27	485	7.8 ± 1.9	Metabolism
Keratin, type I cytoskeletal 19	K1C19	Q9P1Y4	44065/5.04	17	32	344	5.8 ± 1.9	Structural component
Keratin, type I cytoskeletal 18	K1C18	P05783	48029/5.34	17	12	285	5.1 ± 1.7	Structural component
Phosphoglycerate kinase 1	PGK1	P00558	44992/8.30	6	13	73	18.3 ± 4.3	Metabolism
Isocitrate dehydrogenase [NADP] cytoplasmic	IDHC	O75874	46920/6.53	1	3	53	2.6 ± 0.3	Metabolism
Keratin, type II cytoskeletal 8	K2C8	Q96J60	53671/5.52	18	26	273	5.1 ± 1.3	Structural component
60 kDa heat shock protein, mitochondrial	CH60	P10809	61190/5.70	18	23	477	10 ± 3.4	Molecular chaperone
UDP-glucose 6-dehydrogenase	UGDH	O60701	5685/6.73	2	8	75	3.1 ± 1.1	Metabolism
Glutamate dehydrogenase 1	GLUD1	P00367	61701/7.66	4	26	187	8.3 ± 2.6	Metabolism
Aminoacylase-1	ACY1	Q03154	46084/5.77	6	25	246	4.7 ± 1.9	Proteolysis
Glutathione transferase omega-1	GSTO1	P78417	27833/6.24	7	18	264	8.3 ± 2.4	Metabolism
Ubiquitin	UBC	P62988	8560/6.56	7	9	156	2.7 ± 0.8	Proteolysis
Cofilin-1	COF1	P23528	18723/8.22	6	27	306	2.6 ± 1.1	Signal transduction
Acidic leucine-rich nuclear phosphoprotein 32 family member B	AN32B	Q92688	28944/3.94	4	19	136	4.1 ± 1.4	Protein binding
Annexin A3	ANXA3	P12429	36527/5.63	3	12	85	13.5 ± 2.7	Signal transduction
Histone H1.2	H12	P16403	21352/10.94	3	6	54	9.5 ± 2.4	Protein binding
Glyceraldehyde-3-phosphate dehydrogenase	G3P	P04406	36204/8.57	63	59	1299	8.1 ± 2.7	Metabolism
Aldose reductase	ALDR	P15121	36237/6.51	11	25	240	8.5 ± 3.1	Protein binding
Aldo-keto reductase family 1 member C2	AK1C2	P52895	37118/7.13	5	20	229	14.8 ± 4.5	Metabolism
Aldo-keto reductase family 1 member B10	AK1BA	O60218	36230/7.12	3	13	131	13.1 ± 3.8	Metabolism
Complement component 1 Q subcomponent-binding protein, mitochondrial	C1QBP	Q07021	31749/4.74	2	10	78	13.3 ± 3.7	Immune regulation
60S ribosomal protein L6	RL6	Q02878	32766/10.59	11	26	202	11.3 ± 3.7	Transcription regulation
Poly(rC)-binding protein 2	PCBP2	Q15366	38962/6.33	3	13	62	10.2 ± 2.4	Protein binding
Heat shock 70 kDa protein 1	HSP71	P08107	70299/5.48	4	8	115	20.4 ± 4.8	Molecular chaperone
Trifunctional enzyme subunit alpha, mitochondrial	ECHA	P40939	83701/9.16	4	8	98	3.8 ± 1.9	Metabolism
Annexin A6	ANXA6	P08133	76174/5.42	4	3	92	11.4 ± 3.2	Signal transduction
Heat shock 70 kDa protein 1L	HS71L	P34931	70737/5.76	4	6	84	29.5 ± 5.7	Molecular chaperone
Protein disulfide-isomerase A4	PDIA4	P13667	73235/4.96	3	4	81	11.4 ± 3.6	Metabolism
ATP synthase subunit beta, mitochondrial	ATPB	P06576	56525/5.26	14	28	352	22.7 ± 4.9	Metabolism
Pyruvate kinase isozymes M1/M2	KPYM	Q9BWB5	58480/7.96	12	23	334	2.3 ± 1.4	Metabolism
Protein disulfide-isomerase	PDIA1	P07237	57487/4.76	3	5	84	8.8 ± 3.6	Protein binding
Delta-1-pyrroline-5-carboxylate synthetase	P5CS	P54886	88002/6.66	6	6	178	14.3 ± 4.1	Metabolism
Desmoplakin	DESP	P15924	334063/6.44	3	2	64	3.3 ± 1.6	Structural component
ATP-dependent DNA helicase 2 subunit 2	KU86	P13010	83232/5.55	3	7	53	9.6 ± 3.5	Protein binding
Cullin-associated NEDD8-dissociated protein 1	CAND1	Q9P0H7	138029/5.52	6	3	63	4.1 ± 2.1	Transcription regulation
ATP-dependent DNA helicase 2 subunit 1	KU70	P12956	70089/6.23	9	5	107	3.1 ± 1.6	Protein binding
**Down-regulation**								
Protein S100-A6	S100A6	P06703	10231/5.33	2	28	53	3.8 ± 1.4	Calcium ion binding
Calpain small subunit 1	CAPNS1	P04632	28469/5.05	4	13	157	3.1 ± 1.6	Calcium ion binding
Annexin A1	ANXA1	P04083	38922/6.57	2	4	98	2.7 ± 0.8	Calcium ion binding
Interleukin-18 precursor (IL-18)	IL18	Q14116	22597/4.54	12	18	123	2.3 ± 0.6	Immune regulation
Transketolase	TKT	P29401	68531/7.58	9	13	145	6.2 ± 1.9	Metabolism
Profilin-1	PFN1	P07737	15219/8.44	6	21	140	3.4 ± 1.7	Structural component
Pyruvate dehydrogenase E1 component alpha subunit	PDHA1	P08559	43296/8.35	14	8	179	3.5 ± 1.5	Metabolism
Protein SET	SET	Q01105	33469/4.23	2	16	96	4.4 ± 1.7	Signal transduction
Heat shock protein HSP 90-alpha	HS90A	P07900	85013/4.94	40	26	428	2.8 ± 0.5	Molecular chaperone
Histone H2A type 2-A	H2A2A	Q6FI13	14087/10.90	6	36	153	3.7 ± 1.2	Transcription regulation
Poly(rC)-binding protein 1	PCBP1	Q15365	37996/6.66	9	17	118	2.6 ± 1.4	Protein binding
Lamin-A/C	LMNA	P02545	74385/6.57	7	11	86	3.7 ± 1.8	Protein binding

^*a *^For several proteins, a few isoforms were identified in the same individual.

^*b *^Accession numbers were derived from the ExPASy database.

^*c *^Theoretical molecular mass (kDa) and pI from the ExPASy database.

^*d *^Probability-based MOWSE (molecular weight search) scores.

^*e *^Expression change level in HepG2 cells compared with L02 cells.

### Overexpression of PGAM1 in HCC

**Figure 2:**
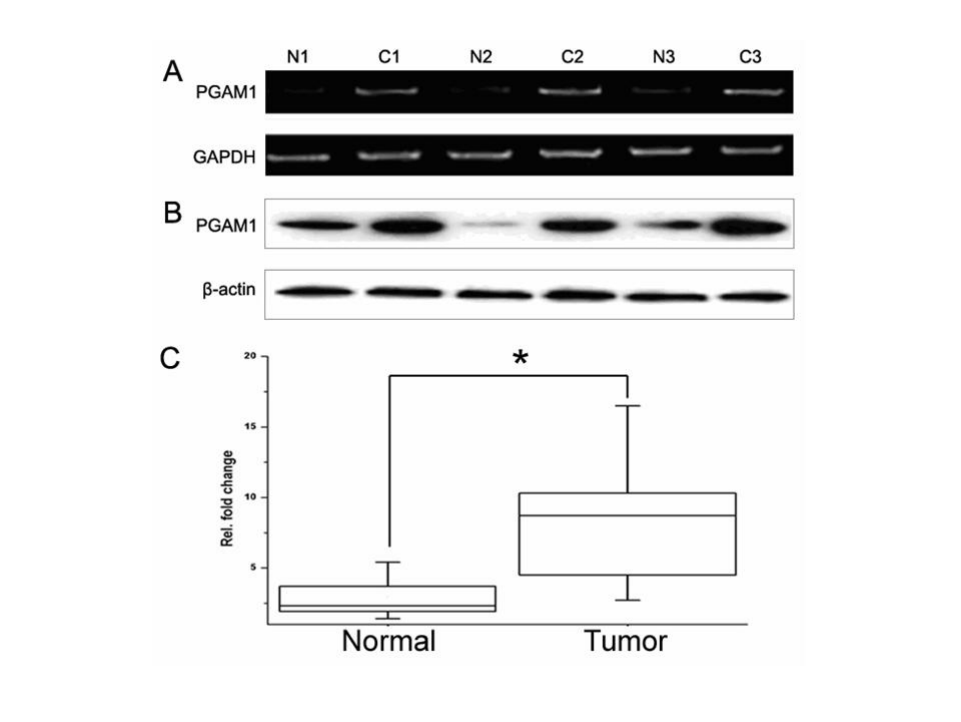
**Overexpression of PGAM1 in HCCs**. (A), expression level of PGAM1 mRNA was semi-quantified by RT-PCR. Pairs of the total mRNA were normalized by GAPDH. (B), representative results of Western blot analysis of HCCs and the adjacent noncancerous samples with β--actin as an internal control. (C), box chart, quantitative Western blot results of HCCs vs. the corresponding normal tissues. To ensure reproducibility, three independent experiments were performed. *, *p *< 0.01, Student's *t *test. *N*, normal liver tissue. *C*, cancer tissue.

To examine if PGAM1 was overexpressed in HCC, a validation experiment was carried out. A total of 54 paired liver cancer tissues were collected, and expression of PGAM1 was compared at both transcriptional and translational levels between HCC tissues and the corresponding adjacent noncancerous tissues. As shown in Fig. 2A, the transcripts of PGAM1 were much higher in HCC, compared with the noncancerous tissues. Further immunoblot analysis was performed using anti-PGAM1 antibody, and overexpression of PGAM1 was observed in 66.7% (36/54) of the HCC tissues (Student's *t *test, *p *< 0.01). Together, our data demonstrated that PGAM1 is overexpressed in HCC tissues at both mRNA and protein levels, which is consistent with the observation in the quantitative proteomic analysis (Fig. 2B and 2C).

### Overexpression of PGAM1 was correlated with poor prognosis of HCC

**Figure 3:**
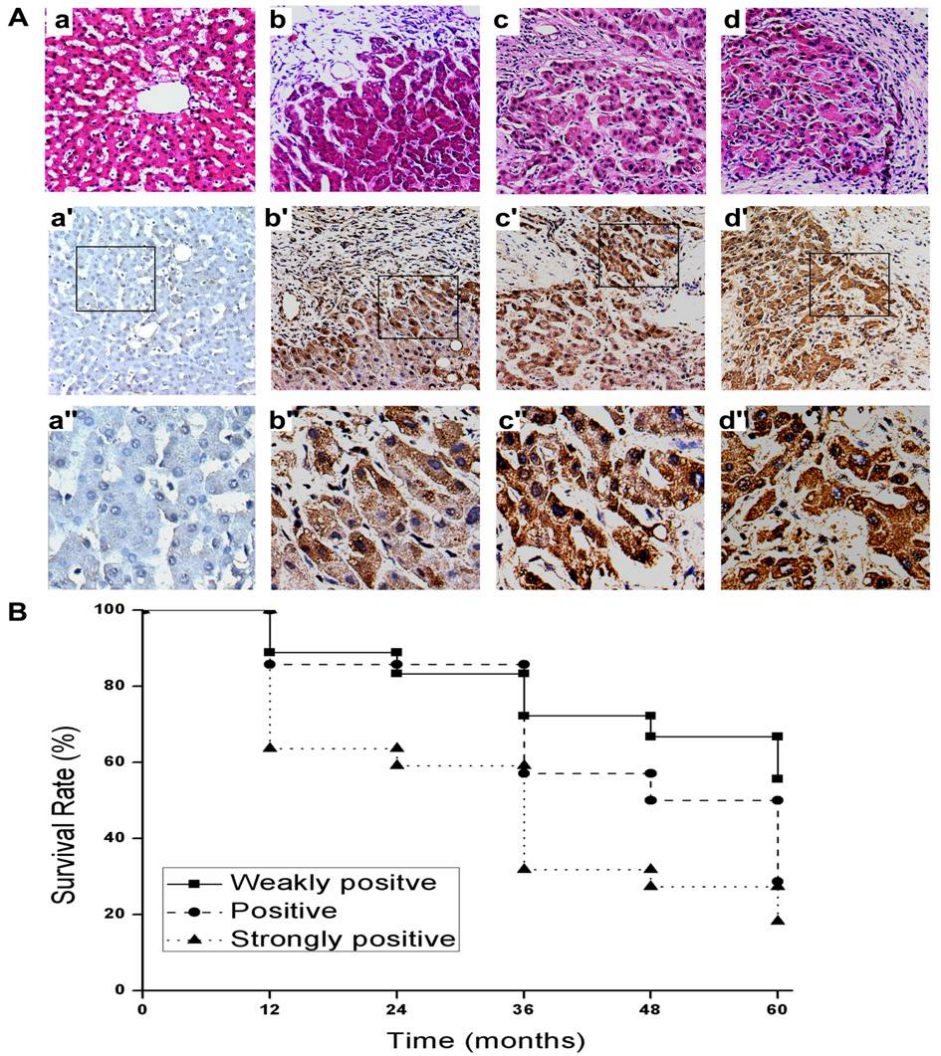
**Immunohistochemical analysis indicated that overexpression of PGAM1 in HCCs was closely associated with the degree of differentiation and the lower survival rates**. (A), staining against PGAM1 confirmed significant differences between normal liver tissues and hepatocellular carcinoma samples more likely to present poor differentiation (*p *< 0.05). a, normal liver tissue; b, well differentiated tissue; c, moderate differentiation; d, poor differentiation. a-d, representative HE staining; a'-d', expression of PGAM1 was examined by immunohistochemistry. a"-d", representative insets of a'-d'. Original magnification, × 200. (B), Kaplan-Meier survival curves showed the correlation between higher levels of PGAM1 expression and lower survival rates (*p *< 0.05).

To further investigate the potential oncogenic properties of PGAM1 in hepatocarcinogenesis, immunohistochemistry was performed to examine PGAM1 expression in paraffin-embedded tissues. 54 pairs of HCC tissues at different clinicopathologic stages and 21 normal liver tissues were prepared for immunohistochemical analysis. Of the 54 HCC samples (mean age, 44.8 ± 8.5 years), 11 were well differentiated, 28 were moderately differentiated, and 15 were poorly differentiated; 14 were in staging I, 21 were in staging II, 9 were in staging III, and 10 were in staging IV according to Surgical Pathologic Staging Criteria (6th Edition, 2002) (Table 1). In 54 paired HCC samples, no or weakly positive staining could be detected in 19 and 81% of non-tumor liver tissues, respectively (Table 3). By contrast, in tumor tissues, weakly positive staining was observed in 24% (13/54), moderately positive staining was about 35% (19/54) and the strong positive staining was 41% (22/54) (Table 4). As shown in Fig. 3A, the staining intensity and the number of positively stained cells were markedly different between normal and hepatoma tissues (*p *< 0.01). Overexpression of PGAM1 was more likely to be present with poor differentiation (*p *< 0.05).

**Table 3:** PGAM1 immunoreactivity in normal liver tissues and hepatocellular carcinoma tissues.

Histodifferentiation Grading	Cases	--	+	+ +	**+ + +**^**a**^	Total Score	**Average Score**^**a**^
Normal liver tissues	21	19% (4/21)	81% (17/21)	0	0	48	2.29 ± 1.02
Hepatocellular carcinoma	54	0	33% (18/54)	26% (14/54)	41% (22/54)	428	7.93 ± 2.58

^a ^Student's t test, *p *< 0.01.

**Table 4:** Relevance of HCC characteristics to PGAM1 immunoreactivity: histodifferentiation to PGAM1.

Histodifferentiation Grading	Cases	--	+	+ +	**+ + +**^**a**^	Total Score	**Average Score**^**b**^
Well differentiated	11	0	73% (8/11)	18% (2/11)	9% (1/11)	63	5.73 ± 2.46
Moderately differentiated	28	0	36% (10/28)	39% (11/28)	25% (7/28)	217	7.75 ± 2.68
Poorly differentiated	15	0	0	7% (1/15)	93% (14/15)	148	9.87 ± 3.17

^a ^Cross χ^2 ^test, *p *< 0.01.

^b^One-way ANOVA analysis, *p *< 0.05; LSD-t test, *p *< 0.05 (well versus moderately; well versus poorly; moderately versus poorly).

To assess the correlation between overexpression of PGAM1 and the survival rates, 54 patients were retrospectively studied (Fig. 3B). The five year survival rates were 55.6%, 28.6%, 18.2% for weakly positive, positive and strongly positive staining samples, respectively. In order to evaluate whether PGAM1 could be utilized as an independent prognostic factor associated with clinical outcome of HCCF, multivariate analyses were carried out using Cox proportional hazard model. The risk variables examined included PGAM1 immunoreactivity (weakly/moderately compared with strongly positive), age of patients (>50 compared with ≤50 years), histodifferentiation (weak/moderate compared with poor differentiation), and surgical pathologic staging (staging I, II, III compared with IV). Our studies suggested that PGAM1 could be developed as an independent prognostic factor for HCC.

### Suppression of liver cancer cell Proliferation by PGAM1-shRNA

In a pilot study, three shRNA expressing plasmids targeting PGAM1 were designed (termed as PGAM1-shRNA-a, b, c), and their silencing effects were evaluated in HepG2 cells. Our data demonstrated that the expression of PGAM1 was remarkably reduced when HepG2 cells were treated with either PGAM1-shRNA-a or PGAM1-shRNA-b whilst no apparent silencing effect could be observed if HepG2 cells were treated with PGAM1-shRNA-c, compared with the negative control shNC (See additional file 1; Fig.S1).

**Figure 4:**
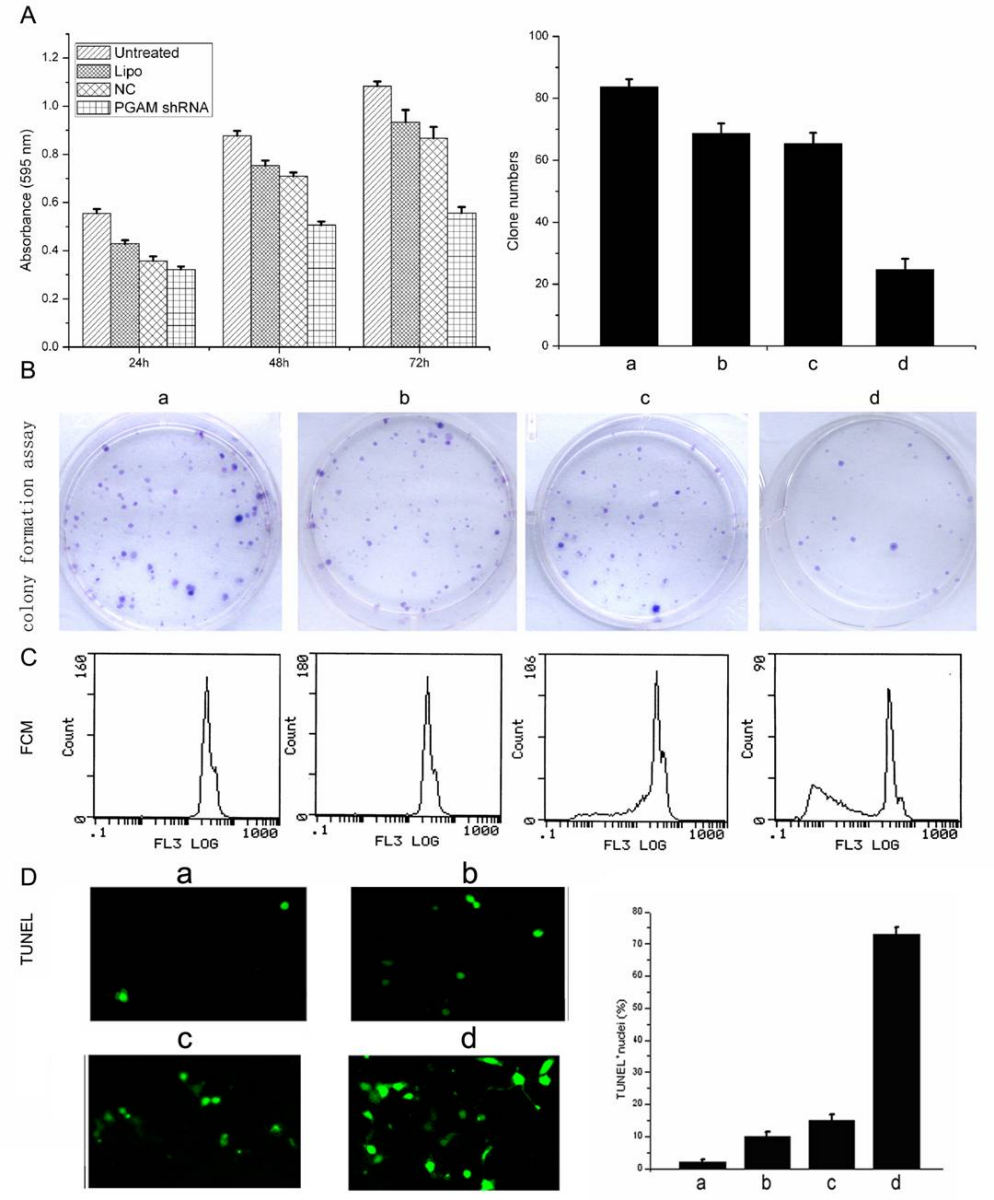
**Suppression of PGAM1 by PGAM1-shRNA-a resulted in cell growth arrest and induced apparent apoptosis *in vitro***. (A) Evaluation of the antiproliferation effects using MTT assay. Inhibition of cell growth by siRNA against PGAM1 was in a duration-dependent manner and the proliferation rate was decreased to 48.6% at 72 h posttransfection. (B) Survival rates of hepatoma cells were examined by colony formation assay. 24 h after transfection, cells were allowed to grow at a density of 300 cells/dish for additional two weeks before staining with Crystal Violet. (C) Flow cytometry analysis was performed and the subdiploid peak increased after 72 h of transfection (*p *< 0.05). (D) Cell apoptosis was assessed by TUNEL assay which showed a remarkably greater percentage of TUMEL positive nuclei of the PGAM1-shRNA-a group vs. the control group. a, untreated; b, Lipofectamine 2000; c, shNC; d, PGAM1-shRNA-a. Results represent the average of three independent experiments and data were shown as mean ± S.D.

To investigate the potential function of PGAM1, the liver cancer cell line HepG2 was treated with PGAM1-shRNA. As shown in Fig. 4A, PGAM1 knockdown by PGAM1-shRNA-a resulted in remarkable inhibition of liver cancer cell proliferation, which was demonstrated by both MTT and clonogenic formation assays. MTT data showed that cell proliferation was suppressed by PGAM1-shRNA-a in duration-dependent manner, and the proliferation ratio was decreased by 48.6% at 72 h posttransfection, compared to the negative control (shNC). In colony formation assay, upon 14-day continuous culture, the clone numbers were 92 ± 3.84, 69 ± 3.38, and 65 ± 4.33 in untreated control, mock control (Lipofectamine 2000), and negative control (shNC), respectively (Fig. 4A). Meanwhile the clone number in the PGAM1-siRNA-a group was 25 ± 3.02 with an inhibition ratio of 72.8% (Dunnett's *t *test, *p *< 0.01) (Fig. 4B).

### Knockdown of PGAM1 expression induced cancer cell apoptosis

To examine if loss of PGAM1 expression induces apoptotic cell death, flow cytometric analysis was performed to measure the sub-G_1 _value of HepG2 liver cancer cell treated with PGAM1-shRNA-a. As shown in Fig 4C, a clear-cut difference was observed at 72 h posttransfection, and the apoptosis/PI positive percentage reached 48.6% for PGAM1-shRNA-a treated cells compared with 1.0%, 1.2% and 7.8% for untreated, Lipofectamine 2000 and HK-shRNA, respectively (*p *< 0.01). As the sub-G_1 _values measured by flow cytometry represent dead cells arising from both apoptosis and necrosis, a more specific TUNEL assay was applied to measure the apoptotic cells induced by PGAM1-shRNA-a. Cell nuclei with DNA strand breaks were revealed by labeling free 3'-OH termini and observed to stain dark green as viewed by fluorescence microscopy, indicating apoptosis, and were recorded as TUNEL-positive nuclei. As shown in Fig. 4D, a significant increase of TUNEL-positive nuclei was observed in the PGAM1-shRNA-a transfected cells (73.7 ± 2.01%), compared with the control groups, 2.4 ± 0.67% (Untreated control), 10.2 ± 1.34% (Lipofectamine 2000), and 15.8 ± 1.67% (NC-shRNA control) (Dunnett's *t *test, *p *< 0.01). Collectively, data obtained from diverse experiments demonstrated that suppression of PGAM1 expression resulted in massive liver cancer cell apoptosis.

To rule out the potential off-target effect, HepG2 cells were treated with another PGAM1 specific shRNA (PGAM1-shRNA-b). As shown in Fig. S2 (A-C) in additional file 1, treatment with PGAM1-shRNA-b in HepG2 cells resulted in remarkable inhibition of cell proliferation, and induction of apoptosis, which were evidenced by the observations from MTT assay, clonogenic formation assay and TUNEL assay.

### PGAM1-shRNA-a inhibited xenograft tumor growth in vivo

**Figure 5:**
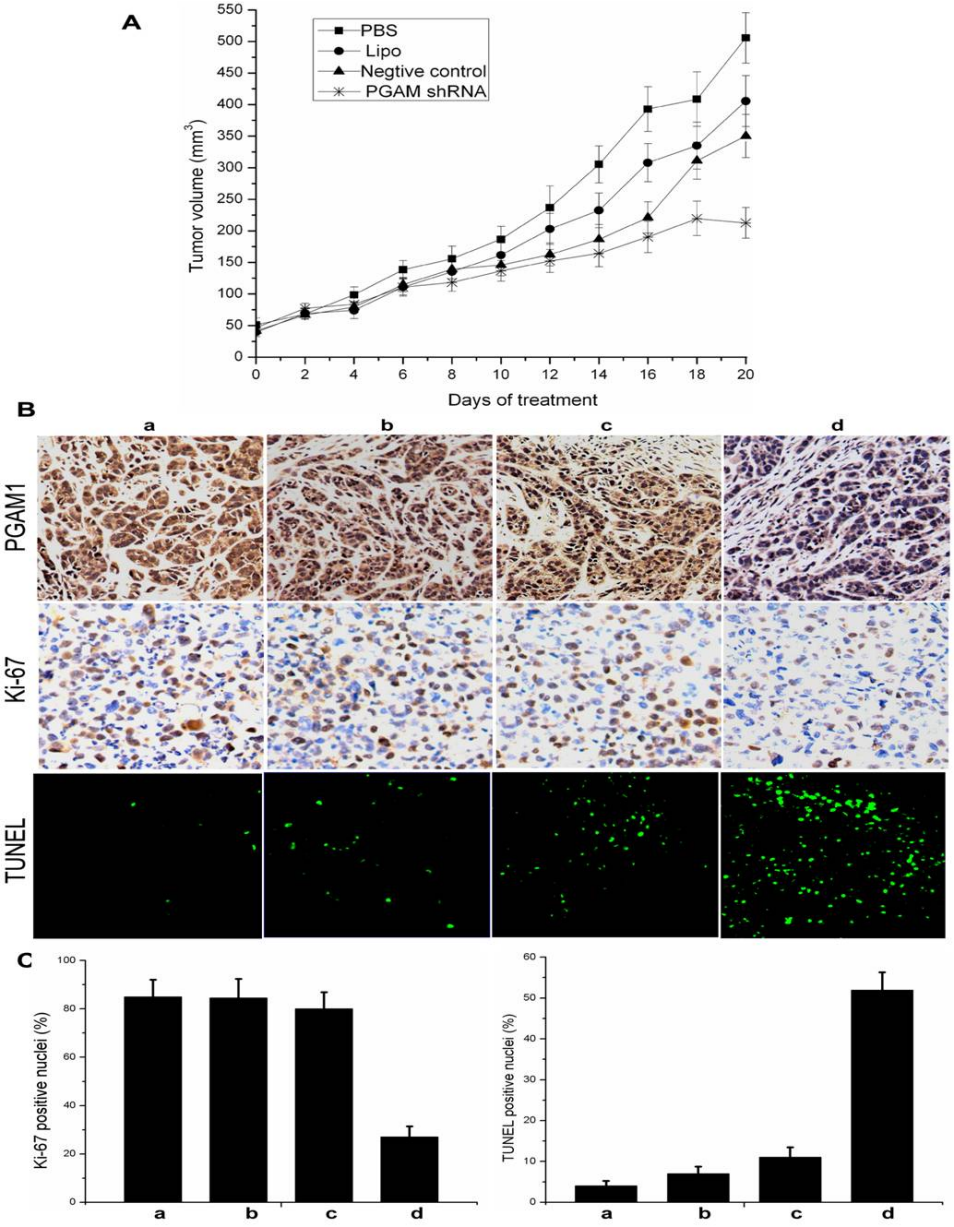
**Treatment with PGAM1-shRNA-a inhibited xenograft tumor growth and induced apoptosis *in vivo***. (A), tumor volume growth curve after tail intravenous injection for 20 days. PGAM1-shRNA-a treatment resulted in significantly decreased tumor growth compared with other control groups (*p *< 0.05). (B) & (C), a significant decrease of PGAM1 expression in tumor-bearing mice examined by immunohistochemical analysis (*upper lane*). Cell proliferation was obviously inhibited and apparent apoptosis was induced (*p *< 0.05). a, PBS; b, Lipofectamine 2000; c, shNC; d, PGAM1-shRNA-a. Data were shown as mean ± S.D.

To extend the above findings, *in vivo *studies were performed using HepG2 xenograft tumor bearing mice. Tumor volumes were measured every 2 days during treatment duration until animals were sacrificed, and no animal death or signs of possible toxicity were observed during this period (data not shown) [34]. Although the tumors of all mice were approximately equal in initial volumes, significant differences in tumor growth were observed upon treatment with PGAM1-shRNA-a. As shown in Fig 5A, the average tumor volumes at the termination of the experiment were 515.65 ± 40.14, 455.58 ± 40.23, and 410.23 ± 34.16 mm^3 ^for PBS, Lipofectamine 2000, and NC-shRNA, respectively (*p *> 0.05). In comparison, tumor volumes in mice treated with PGAM1-shRNA-a were 212.71 ± 24.28 mm^3^, which were on average over 58.7% smaller than those in controls treated with PBS (Student's *t *test, *p *< 0.01) (Fig. 5A). To validate PGAM1-shRNA-a mediated suppression of PGAM1 expression, immunohistochemical analysis against anti-PGAM1 antibody was performed to detect PGAM1 expression level in the tumor-bearing mice which had been subjected to PGAM-shRNA treatment. As shown in Fig. 5B, tail intravenous injections of PGAM-shRNA-a resulted in more than 75% suppression of PGAM1 in tumor-bearing mice, whilst no obvious difference could be observed regarding the expression level of PGAM1 in the control mice either treated with Lipofectamine 2000 or NC-shRNA, relative to injection with PBS.

As PGAM1 repression inhibited cancer cell growth and induced remarkable apoptotic cell death *in vitro*, we have particular interest to examine the potential function underlying PGAM1-shRNA-a mediated anti-tumor activity *in vivo*. To this end, tumor cell proliferation and apoptosis were assessed by Ki-67 immunoreactivity analysis and TUNEL assay. As shown in Fig. 5B, Ki-67 positive nuclei were decreased over 68% for control with PBS (Student's *t *test, *p *< 0.01). In contrast, TUNEL assay showed a remarkably higher percentage of TUNEL positive nuclei in PGAM1-shRNA-a treated group, relative to injection of PBS control. Our data suggested that suppression of PGAM1 expression mediated by PGAM1-shRNA-a could significantly inhibit cell proliferation and induce apoptosis *in vivo*.

## Discussion

Hepatocellular carcinoma (HCC), one of the most common malignancies worldwide, remains a major health problem with increasing incidence rates even to date [4,35], and there is an urgent need to identify novel molecular targets for diagnosis, prognosis and treatment of HCC. In the current study, a SILAC-based quantitative proteomics approach was applied to profile the altered expressed proteins between HepG2 cells and L02 cells, resulting in identification of 63 distinct proteins with altered expression, which were associated with cell metabolism, proliferation and/or apoptosis. Among them, Profilin1, a member of profilin family, also known as PFN1, was ubiquitous and down-regulated more than 3-fold in HepG2 cells. As a tumor suppressor in breast cancer cells, PFN1 was reported to be involved in multiple cell behaviors, including cell adhesion, growth, proliferation and signal transduction [36,37]. On the contrary, some key enzymes participated in glycolytic pathway were overexpressed in HepG2 cells, exemplified by enolase, which catalysed the conversion of 2-phosphoglycerate to phosphoenolpyruvate; Phosphoglycerate kinase 1 was overexpressed more than 18-fold which catalysed the conversion of 1,3-bisphosphoglycerate to 3-phosphoglycerate coupled with the generation of ATP. Most intriguingly, we found that phosphoglycerate mutase 1 (PGAM1) was shown an upregulation up to 6-fold. As an enzyme in glycolysis, PGAM1 was ubiquitously expressed in *human*, *Bacillus stearothermophilus *[38], *Escherichia coli *[39], *Entamoeba histolytica *[40], *et al*, functions to catalyze the interconversion of 3-phosphoglycerate and 2-phosphoglycerate with 2,3-bisphosphoglycerate (2,3-BPG) [15,41]. A recent study revealed that PGAM1 was overexpressed in breast cancer, and suppression PGAM1 expression displayed a profound antiproliferative effect, underscoring its important role in carcinogenesis [14]. Obviously, more extensive investigations on the functions of PGAM1 which was upregulated in HCC are required to elucidate the role of PGAM1 in hepatocarcinogenesis.

**Figure 6:**
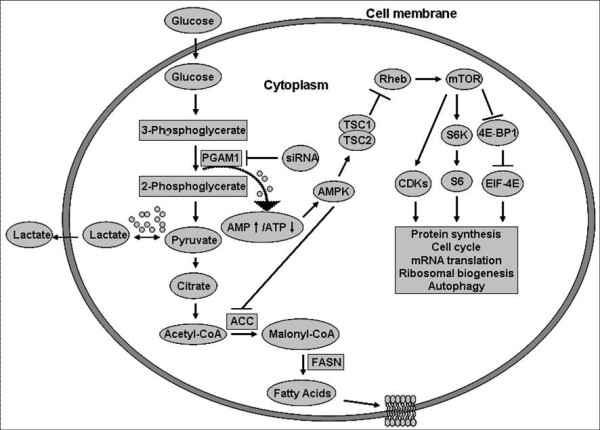
**A model of glycolysis illustrating the possible involvement in cellular energy signaling pathways**. Arrows represent activation, whereas bars represent inhibition. Dotted arrows indicate the increased ratio of AMP/ATP. Small circles represent ATP. AMPK, AMP-activated protein kinase; TSC, tuberous sclerosis complex; Rheb, Ras homologue enriched in brain; mTOR, mammalian target of rapamycin; S6K, ribosomal protein S6 kinases; 4E-BP1, eIF4E-binding protein; ACC, acetyl-CoA carboxylase; FASN, fatty-acid synthase; CDKs, cyclin dependent kinases.

As an intracellular hallmark of neoplasm, the increased level of glycolysis enables cancer cells to survive despite the poor conditions [42]. Fifty years ago, Otto Warburg had demonstrated that cancer cells were oxygen-independent for producing ATP, particularly in the hypoxic tumor microenvironment [9,43]. Previous studies demonstrated that hypoxia inducible factor (HIF) enhanced glycolysis by increasing the transcription of glycolytic enzyme genes to protect cancer cells from energy starvation [44,45]. It has been clear that, highly proliferative cancer cells need to synthesize fatty acids *de novo *to continually provide lipids for membrane production. An increased glycolytic flux could lead to an augmented amount of metabolic precursors for the synthesis of nucleic acid, amino acid or lipid which are essential for the cancer cell growth and proliferation [46,47]. Conversely, inhibition of glycolytic pathway results in decreasing not only amino acid and lipid synthesis but also ATP production. An increased AMP/ATP ratio is important for activation of AMP-activated protein kinase (AMPK). Once activated by energy starvation, AMPK directly phosphorylates tuberous sclerosis complex 2 (TSC2) on T1227 and S1345, stimulates its GTPase activity resulting in the inhibition of Ras homologue enriched in brain (Rheb) which is essential for mammalian target of rapamycin (mTOR) activity. Moreover, inactivation of mTOR was strongly correlated with cell growth arrest and apoptosis [12]. On the other hand, acetyl-CoA carboxylase (ACC) is an important rate-controlling enzyme for the synthesis of malonyl-CoA, which is not only a critical precursor for biosynthesis of fatty acids but also a potent inhibitor of mitochondrial fatty acid oxidation. In this case, phosphorylation and inhibition of ACC by AMPK leads to a fall in malonyl-CoA content and a subsequent decrease in triglyceride synthesis concomitantly with an increase in β-oxidation [48]. In general, it has been considered that glycolysis plays a pivotal role for ATP production and cell growth in transformed cells (Fig. 6). Considerable effort has been made to elucidate the close correlation between rates of aerobic glycolysis and the degree of malignancy [49]. In view of this, the decreased of glucose level should be strongly tumoricidal for transformed cells proliferation [50]. For example, 3-bromo pyruvate, an inhibitor of hexokinase, has been demonstrated to inhibit glycolysis and effectively kill hepatoma cells in tissue culture even at a lower concentration [51]. Furthermore, in the presence of GAPDH, Nm23-H1 could phosphorylate PGAM1 and inhibit PGAM1 activity resulting in suppression of glycolysis and inducing growth arrest in various cancer cells, including glioblastoma cell line Tx3095, small lung cancer cell line GLC4, beast carcinoma cell lines MCF-7 and MDA-MB-453, etc [13]. Under this circumstance, PGAM1 should be a potential diagnostic biomarker, as well as a therapeutic target for various malignancies.

Clinico-pathological analysis indicated that overexpression of PGAM1 was associated with 66.7% HCC, and strongly correlated with poor differentiation and decreased survival rates (*p *< 0.01). Our studies suggested that PGAM1 has the potential to be developed as a useful diagnostic and prognostic marker for HCC. Further studies should be performed to evaluate if PGAM1 could be utilized as an independent biomarker for early diagnosis of HCC. On the other hand, silencing expression of PGAM1 significantly induced liver cancer cell apoptosis both *in vitro *and *in vivo*. Apoptosis is a major barrier that must be circumvented during malignant transformation. Cancer cells evolve to evade apoptosis so that they can escape from being cleared away by the surveillance system and can survive in the crucial tumor microenvironment, such as hypoxia and nutrition depletion [52]. Defective apoptosis was considered as a major causative factor in the genesis and development of many human cancers, triggering tumor selective apoptosis in cancer cells exploited into a promising strategy for clinical treatment [53]. The strong apoptosis-promoting activities mediated by PGAM1-siRNA suggested that PGAM1 would be an attractive drug target for therapeutic treatment with HCC. Further intensive studies should be conducted to pinpoint the molecular mechanisms underlying PGAM1-siRNA mediated cell death.

## Conclusions

So far, a small but increasing number of reports were documented regarding targeting a cellular metabolic enzyme for cancer therapy [54], thus the current study provided new insights for potential clinical treatment of HCC using siRNA-mediated suppression of PGAM1 expression since RNAi technology has emerged as a powerful tool to silence gene expression in mammalian cells so that it could be used to investigate the gene function [55]. Hopefully, shRNA-mediated suppression of PGAM1 expression, combined with conventional surgical resection and chemotherapy strategies, will open a new avenue for clinical treatment of hepatocellular carcinoma.

## Competing interests

The authors declare that they have no competing interests.

## Authors' contributions

CHH and YQW conceived of the study; FLR, RL, YLL and YZ and LJC carried out the proteomics studies; HW and HYZ and FLR collected samples and participated in prognosis analysis; XCC, DQZ, HW and FLR were involved in the pathology analysis; APT assisted in molecular experiments; FLR and RL performed the statistical analysis, and FLR, RL, and CHH drafted the manuscript; All authors have read and approved the final manuscript.

## Supplementary Material

Additional file 1 **A brief description of PGAM1-shRNA strategy**. There are one table (Table S1) and two figures (Fig.S1 and Fig.S2) in the additional file 1. The sequences of PGAM1-shRNA-a, -b and -c were listed in Table S1. Fig. S1 showed the effect of PGAM1-shRNA-a, -b, -c on suppression of PGAM1 expression in HepG2 cells. Fig. S2 elaborated on the PGAM1-shRNA-b induced inhibition of HepG2 cell proliferation and induction of apoptosis *in vitro *to rule out the potential off-target effect. Click here for file
