# Supplementary material for: Quantitative proteomics identification of phosphoglycerate mutase 1 as a novel therapeutic target in hepatocellular carcinoma
Source: Mol Cancer. 2010 Apr 19;9:81. doi: 10.1186/1476-4598-9-81 (PMC2873438; doi:10.1186/1476-4598-9-81)
Supplement: Additional file 1 — A brief description of PGAM1-shRNA strategy. There are one table (Table S1) and two figures (Fig.S1 and Fig.S2) in the additional file 1. The sequences of PGAM1-shRNA-a, -b and -c were listed in Table S1. Fig. S1 showed the effect of PGAM1-shRNA-a, -b, -c on suppression of PGAM1 expression in HepG2 cells. Fig. S2 elaborated on the PGAM1-shRNA-b induced inhibition of HepG2 cell proliferation and induction of apoptosis in vitro to rule out the potential off-target effect. [file 1476-4598-9-81-S1.DOC]

**Table S1.** The sequences of PGAM1-shRNA-a, -b and –c (See Methods).

| **PGAM1-shRNA-a**  Sense: 5’-CACCGGTGAAGATCTGGAGGCGCTTCAAGAGAGCGCCTCCAGATCTTCACCTTTTTTG-3’  Antisense:  5’-GATCCAAAAAAGGTGAAGATCTGGAGGCGCTCTCTTGAAGCGCCTCCAGATCTTCACC-3’ |
| --- |
| **PGAM1-shRNA-b**  Sense: 5’-CACCGGGTCTAACCGGTCTCAATAATTCAAGAGATTATTGAGACCGGTTAGACCCTTTTTTG-3’  Antisense: 5’-GATCCAAAAAAGGGTCTAACCGGTCTCAATAATCTCTTGAATTATTGAGACCGGTTAGACCC -3’ |
| **PGAM1-shRNA-c**  Sense:  5’-CACCGGTCTCAATAAAGCAGAAACTTTCAAGAGAAGTTTCTGCTTTATTGAGACCTTTTTTG-3’  Antisense:  5’-GATCCAAAAAAGGTCTCAATAAAGCAGAAACTTCTCTTGAAAGTTTCTGCTTTATTGAGACC -3’ |

**Fig. S1. The effect of PGAM1-shRNA-a, -b, -c on suppression of PGAM1 expression.** The HepG2 cells were transfected with PGAM1-shRNA-a, -b, -c and shNC, respectively. Expression of PGAM1 was determined by semi-quantitative RT-PCR (A), and by immunoblot (B). Lane a, Untreated control; Lane b, Transfection reagent Lipofectamine 2000 control; Lane c, shNC control; Lane d, PGAM1-shRNA-c; Lane e, PGAM1-shRNA-b; Lane f, PGAM1-shRNA-a.

**
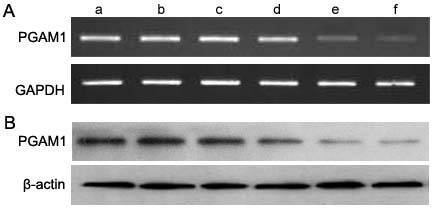
**

**Fig. S2. Suppression of PGAM1 expression mediated by PGAM1-shRNA-b induced remarkable inhibition of HepG2 cell proliferation, and induction of apoptosis *in vitro*.** (A) Cell proliferation was measured by MTT assay. The proliferation rate was decreased to 47.4% at 72 h posttransfection. (B) Apoptosis was examined by TUNEL assay. HepG2 cells treated with PGAM1-shRNA-b showed a greater percentage of TUMEL positive nuclei, compared with the control group. a, Untreated control; b, Transfection reagent Lipofectamine 2000 control; c**,** shNC control; d, PGAM1-shRNA-b. (C) Cell Survival was assessed by colony formation assay. a, Untreated control; b, Transfection reagent Lipofectamine 2000 control; c**,** shNC control; d, PGAM1-shRNA-b. Results represent the average of three independent experiments and data were shown as mean ± S.D.

**
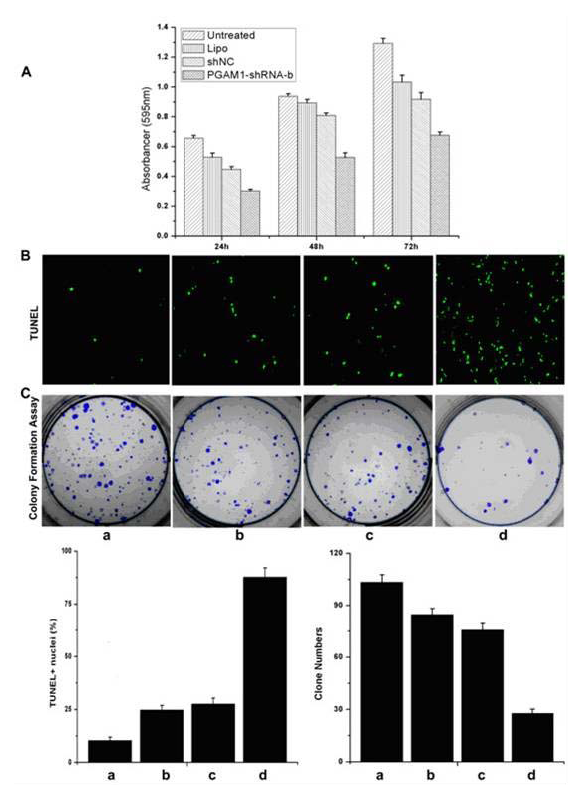
**
